# Climate and Human Pressure Constraints Co-Explain Regional Plant Invasion at Different Spatial Scales

**DOI:** 10.1371/journal.pone.0164629

**Published:** 2016-10-14

**Authors:** Juan Antonio Campos, Gonzalo García-Baquero, Lidia Caño, Idoia Biurrun, Itziar García-Mijangos, Javier Loidi, Mercedes Herrera

**Affiliations:** 1 Department of Plant Biology and Ecology, University of the Basque Country (UPV/EHU), Bilbao, Spain; 2 Ikerbasque, Basque Fundation for Science, Bilbao, Spain; Fudan University, CHINA

## Abstract

Alien species invasion represents a global threat to biodiversity and ecosystems. Explaining invasion patterns in terms of environmental constraints will help us to assess invasion risks and plan control strategies. We aim to identify plant invasion patterns in the Basque Country (Spain), and to determine the effects of climate and human pressure on that pattern. We modeled the regional distribution of 89 invasive plant species using two approaches. First, distance-based Moran’s eigenvector maps were used to partition variation in the invasive species richness, S, into spatial components at broad and fine scales; redundancy analysis was then used to explain those components on the basis of climate and human pressure descriptors. Second, we used generalized additive mixed modeling to fit species-specific responses to the same descriptors. Climate and human pressure descriptors have different effects on S at different spatial scales. Broad-scale spatially structured temperature and precipitation, and fine-scale spatially structured human population density and percentage of natural and semi-natural areas, explained altogether 38.7% of the total variance. The distribution of 84% of the individually tested species was related to either temperature, precipitation or both, and 68% was related to either population density or natural and semi-natural areas, displaying similar responses. The spatial pattern of the invasive species richness is strongly environmentally forced, mainly by climate factors. Since individual species responses were proved to be both similarly constrained in shape and explained variance by the same environmental factors, we conclude that the pattern of invasive species richness results from individual species’ environmental preferences.

## Introduction

Invasion by alien species and climate change are two of the main global threats to biodiversity [1, 2] and ecosystem services [3]. Additionally, since plant invasion dynamics is known to be highly responsive to rising temperature, altered precipitation, and various disturbances associated with changes in land use [4, 5], recent research has suggested increased invasion risk in a global change scenario [6, 7].

Identifying invasion patterns and hotspots, i.e. areas that host the highest numbers of invasive species, and the main factors associated with invasion at regional or larger scales [8], is a highly valuable tool for ecologists and managers in order to better target eradication and control. Explanatory distribution models based uniquely on climatic factors provide useful knowledge by identifying the climatic constraints to the spread of alien species [5]. However, these models assume that the introduction effort (propagule pressure) and the level of disturbance might be comparable across regions. Propagule pressure is closely related to human activity [9, 10], which is usually assessed through various surrogates such as human population density. Likewise, the structure of the landscape, commonly determined in Europe by human activities, is known to influence the dispersal and establishment of invasive plant species [11–13]. Consistently, many research works have reported significant relationships between land cover descriptors and patterns of specific richness (e.g., [14, 15]). For this reason, including human pressure-related predictors such as population density or land cover predictors is likely to improve the accuracy of explanatory models [16]. Moreover, it has been argued that the environmental control of the spatial distribution of species responds to a hierarchical scheme in which climatic variables change at the largest spatial scales, whereas landscape descriptors vary at smaller scales [16, 17].

In this work we first aim to model the relationship between the invasive plant richness and climate and human pressure predictors in the Basque Country region (Spain). In this region, 20.8% of the vascular flora (487 species) is non-native, of which 89 (i.e. 18.6% of the alien species) are considered invasive according to Richardson *et al*. (2000) [18]. The Basque Country region is a suitable model system for testing for the joint effect of climate and human pressure descriptors on invasive species richness distribution since two differentiated bioclimates (Temperate and Mediterranean) coexist in a relatively small territory and the landscape consists of a mosaic of natural, semi-natural and urban areas. Species richness itself, however, cannot be assumed to be characterized by an environmental niche (but see [19]). For this reason, the existence of invasion hot spots should be the outcome of the fact that a high number of invasive species display similar favorable environmental niches. By addressing the individual response of a set of frequent invasive species distribution to environmental predictors we can obtain complementary information on the process driving invasive species richness distribution patterns.

Therefore, in this research, besides modeling the relationship between the invasive plant richness and climate and human pressure predictors, we intend to model the species-specific response of the most frequent species to the same predictors. Hence we address the following questions: (1) Do climate and human pressure constraints explain the spatial pattern of invasive species richness, and if so, how? (2) Do species-specific responses reflect the existence of commonly preferred environmental settings for invasive plants? (3) If affirmative, do these species-specific environmental preferences match the spatial pattern of invasive plant species richness?

## Material and Methods

### Study area

**Fig 1:**
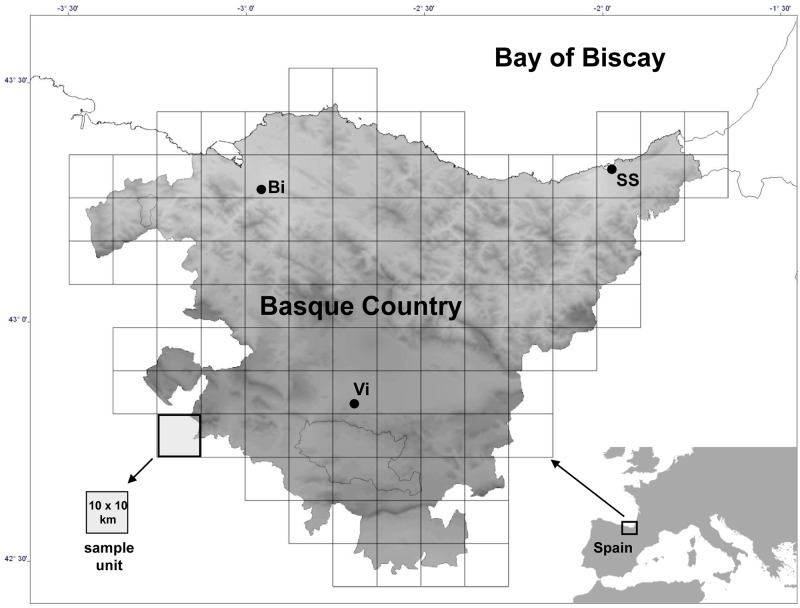
Location of the Basque Country (study area) in the Iberian Peninsula. Main cities: Bi (Bilbao), SS (San Sebastián) and Vi (Vitoria).

The Basque Country region (Spain) occupies 7234 km^2^ in the northern Iberian Peninsula, with its approximate geographic center at 43°02’N, 02°30’W (Fig 1). The main climatic gradient has a north-south direction and, as distance from the sea increases, winter temperature and rainfall decrease. Across a south-north distance of only 120 km, the range in mean annual temperature is 9°–14°; the range in annual precipitation is 600–2400 mm (S1 Fig). The area is divided into two bioclimatic zones: A temperate climate prevails in the north and a Mediterranean climate is restricted to the southern end.

Forest plantations (mainly in the northern half) and agricultural land (mainly in the south) occupy 29% and 13.5% of the Basque Country, respectively. Natural forests (27.7%), grasslands and shrublands (11.2%), meadows (13.3%), artificial areas (5.5%), and wetlands and coastal habitats (1%) occupy the remaining territory. The human population is concentrated around the three main cities, mostly in the Bilbao (42% of the total population) and San Sebastián (21%) metropolitan areas (Fig 1 and S1 Fig).

### Data compilation and preparation

We used data on the distribution of 89 species (S1 Table) that were considered to be invasive plants in the Basque Country [20], defined as “alien plants that produce reproductive offspring, often in very large numbers, at considerable distances from parent plants (…), and thus have the potential to spread over a considerable area” [18].

**Fig 2:**
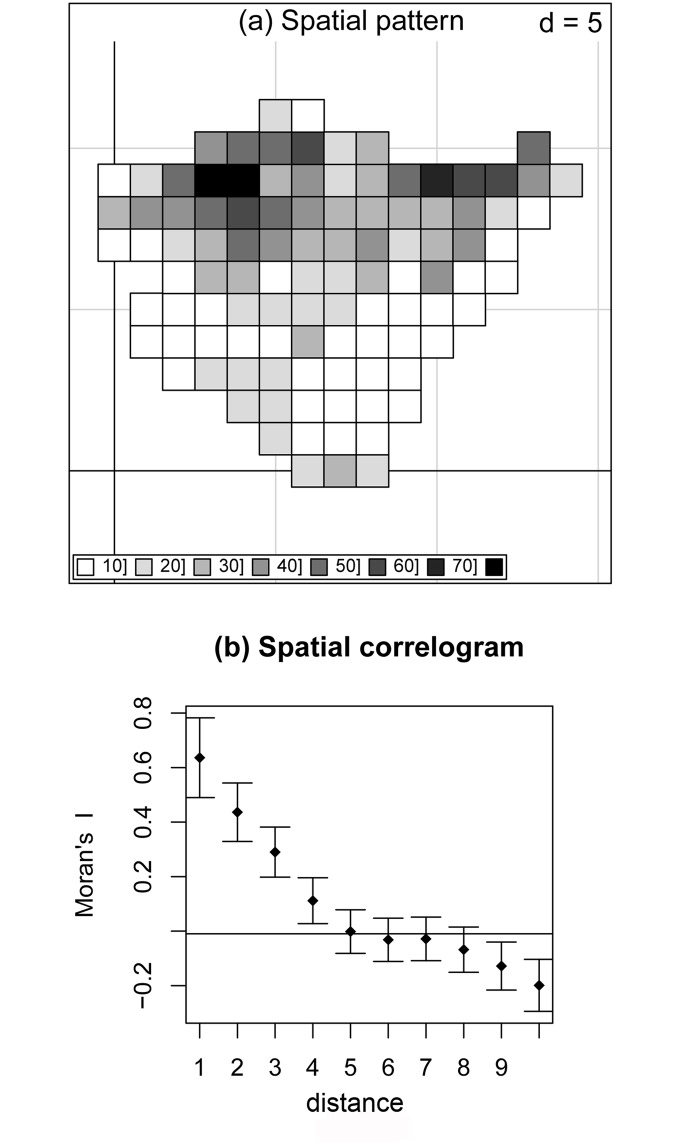
Spatial pattern of S, the number of invasive alien plant species per 10 km x 10 km UTM cell in *n* = 104 cells (a) and spatial correlogram based on Moran’s I statistic for S (b). The plot displays the spatial correlation values against distance (1: 0–10 km, 2: 10–20 km, etc.). Distance (d) is in units of 10 km.

We assembled a three-matrix data set. The first was a species composition matrix of 104 sample units (of 10 km x 10 km UTM cells) x 89 invasive alien plants, where each element represented the presence/absence within a cell. The invasive species *richness* (S), or the number of invasive alien plant species per UTM cell (Fig 2a), was derived from this matrix. The data contained in this matrix were obtained by integrating 18,224 citations, herbarium specimens and species observation records from vegetation surveys compiled in the BIOVEG vegetation plot database [21], corresponding to the period 1970–2009. The invasive species included in the study are known to have been widespread throughout the territory during the last 50 years [20] and all recorded populations were assumed to have persisted throughout the survey period.

The second matrix was an environmental matrix of the same 104 sample units x 5 environmental descriptors. These descriptors (S2 Fig) were: mean annual temperature in °C and annual precipitation in mm (climate descriptors), human population density (per 100 km^2^) and percentage of natural and semi-natural areas in a cell (human pressure), and grain (cell) surface in km^2^. The last variable was included for later use as a covariate to control for potential effects of differing grain surface for cells located in the administrative limits of the Basque Country. The climatic descriptors were calculated from the Digital Climatic Atlas of Spain [22], based on data for the period 1955–1999. Although many other climatic descriptors and indexes (such as summer and autumn rainfall, continentality index, thermicity index, mean minimum and maximum temperature in January, etc.) were initially considered (Table 1), they were all eventually discarded due to high collinearity (Kendall's tau > 0.75) with annual temperature and precipitation.

**Table 1:** Numerical summaries of the response and descriptor variables.

Variable	Description	Range	Mean	StDev
S	Species number (species per 100 km^2^)	79–0 = 79	20	18
T*	Mean annual temperature (°C)	14.0–9.7 = 4.3	12.0	1.1
P*	Annual precipitation (mm)	2272–546 = 1746	1219	365
T_min_	Mean January temperature (°C)	8.5–2.9 = 5.6	5.6	1.5
T_max_	Mean July temperature (°C)	21.9–17.4 = 4.5	19.1	0.7
CI	Continentality index (T_max_—T_min_ in °C)	16.1–11.2 = 4.9	13.5	1.3
Io_July_	P_July_ / T_July_	6.7–1.3 = 5.4	2.9	1.0
Ios2	(P_July_ + P_August_) / (T_July_ + T_August_)	6.9–1.2 = 5.7	3.2	1.1
T_summer_	T_June_ + T_July_ + T_August_	62.4–49.3 = 13.1	54.8	2.3
P_summer_	P_June_ + P_July_ + P_august_	387.4–96.9 = 290.5	195.7	60.6
PopDen*	Human population density (per 100 km^2^)	4183–0 = 4183	310.0	638.0
NaturPerc*	Percentage of natural and semi-natural areas (%)	93.4–9.3 = 84.2	45.2	22.2
AgricPerc	Percentage of agricultural (incl. lands devoted to forestry) areas (%)	86.6–0.3 = 86.3	40.2	25.3
InfraPerc	Percentage of infrastructural (incl. roads) areas (%)	39.7–0.0 = 39.7	4.1	6.9

The four climate and human pressure descriptors used in this work as explanatory variables are marked with an asterisk (*). The other nine variables that were initially considered as potential descriptors were discarded due to high collinearity with other descriptors (see text for details). S (response variable) has been mapped in Fig 2; in addition, see maps and correlograms of the four environmental descriptors in figures of Supporting Information (S1 and S3 Figs).

Similarly, to consider the human pressure descriptors, we used the EUNIS map of the Basque Country [23] to calculate the percentage of natural and semi-natural areas and the official census of 2005 to compute the human population density (National Institute of Statistics). While other landscape descriptors relative to agricultural and urban lands were initially considered (Table 1), they were discarded due to high collinearity with human population density and percentage of natural and semi-natural areas. All the GIS procedures involved in the setting up of the environmental descriptors were performed using the software MiraMon [24]. The third matrix was a spatial matrix of the same 104 sample units (of 10 km x 10 km UTM cells) x 2 (*X*, *Y*) UTM coordinates. Hence the typical size of grain cell (Fig 1 and S2 Fig) is 100 km^2^ (10 km x 10 km); the sampling interval, as distance between centroids of the grid cells, is 10 km between neighboring sampling units; and the extent or range is about 10,000 km^2^. The full data set is available in S1 File.

### Data analysis

#### Modeling the relationship between the richness of invasive alien plant species (S) and climate and human pressure descriptors

To test for spatial autocorrelation in the number of invasive alien plant species per UTM cell (invasive plant richness, S), we computed a spatial correlogram based on Moran’s I statistic [25]; the Holm correction [26] was applied to decrease the risk of type I errors. Similar correlograms were computed for all four environmental variables (S3 Fig).

To model the relationship between the response variable S and the environmental descriptors, we applied the following procedure, which can be replicated using the full R coding provided in S2 File. In the first step, we constructed orthogonal spatial variables representing structures at multiple scales, for which the principal coordinates of neighbor matrices (PCNM) method [27, 28], a distance-based class of the Moran’s eigenvector maps family or dbMEM [29], were used. As a result, we obtained *n*-1 = 103 orthogonal eigenvectors (= PCNM spatial variables) describing both positive (52 eigenvectors) and negative (51 eigenvectors) spatial correlation. Since negative spatial correlation is of interest mainly for modeling biotic interactions [30] and not for modeling environmentally-induced spatial variation in species composition (for which eigenvectors describing positive correlation are commonly used), in this analysis we selected and used only the 52 orthogonal eigenvectors representing positive spatial correlation.

In the second step, these eigenvectors were tested against the response S using redundancy analysis and forward selection with a double stopping criterion [31]. Redundancy analysis (RDA) is a technique for multivariate (multi-response) or univariate regression [30] analysis in either one- or two-dimensional spatial settings [28]. As a result, a set of 16 significant PCNM spatial variables was selected (S4 Fig). These significant spatial variables represented spatial structures in S at different spatial scales.

In the third step, this set of PCNM variables specifying the spatial component in S was divided, according to the size of the patterns ([30], Chapter 13—Spatial analysis), into two subsets. One of these subsets (five PCNMs) constituted the broad-scale spatial component; the other (nine PCNMs), the fine-scale spatial component. Therefore, five PCNMs were used to model spatial variation at a broad scale and nine other PCNMs were used to model spatial variation at a fine scale. Whereas these choices are somewhat arbitrary in the sense that “*no universal rule defining what is broad- and fine-scale has been proposed yet*” [30], we observed no change in the conclusions of the analysis when the above two subsets were defined in slightly different ways (i.e. using subsets of four and ten or six and eight PCNMs, instead of five and nine, to define broad-scale and fine-scale components, respectively).

The number of PCNM variables used in this work is comparable with (or even less than) the number of PCNMs used in previous research. For example, Borcard et al. (2004) [28] used 50 PCNMs to model the abundance of *Adiantum tomentosum* measured in 260 sampling units; in the same paper, they used 12 PCNMs to model chlorophyll measured in 63 sites in a French lagoon. In the fourth step, after double-checking the significance of the PCNM spatial variables at the two different spatial scales, we used the corresponding RDA canonical axes to extract spatial structures in S. As a result, we obtained one RDA canonical axis modeling broad-scale spatial structures in S, and another one modeling fine-scale spatial structures. These axes are quantitative continuous random variables taking positive and negative values. In the fifth and last step, we tested for environmental forcing in S. To do this, the two significant canonical axes (representing fine- and broad-scale spatial variation) were regressed, in two separate regressions, on all climate and disturbance descriptors, for which we used a normal regression model with canonical (identity) link function, while controlling for potential effects of differing grain surface. A graphical schematic description of PCNM analysis was published by Borcard et al. (2004) [28] in Figure A1 of their Appendix A.

#### Species-specific responses to climate and human pressure descriptors

We used Generalized Additive Mixed Models (GAMMs) [32, 33] to model relationships between the occurrence of (individual) species and the four selected environmental variables without assuming linearity. Tests were restricted to 37 invasive alien species with relative frequency > 0.2. In order to guarantee the assumption of independence and hence obtain correct tests and estimates, spatial autocorrelation [34] in model residuals was simultaneously modeled [33]. The appropriate spatial autocorrelation structures [35] to account for spatial autocorrelation in model residuals were identified, species by species, using semivariograms [36] that were implemented with the function Variogram() of the package nlme [37]. Once identified, these structures were incorporated [33] into the basic models (i.e. models that, at that stage, had only smoother terms), which were refitted using the function gamm() of the R package mgcv [38]. This function not only permits mixed modeling, but also finds out automatically the right amount of smoothing. Species grouping based in life forms (annuals vs. perennials) and in the broadest features of phylogeny (eudicots vs. monocots) was also explored. All statistical analyses were performed using R software [39] and the complete R coding used for the analysis is available in S2 File. Together with S1 File, this R code allows the full replication of our statistical analysis.

## Results

### Spatial pattern of alien plant invasion in the Basque Country

Our data show a sharp contrast in the pattern of the number (per UTM cell) of invasive alien plant species, S (Fig 2a). The northern coastal areas are the most invaded, with 30–70 species per cell, and present the highest registered values in the two main urban areas (Bilbao and San Sebastián). In contrast, the southern Mediterranean areas registered the lowest S values (0–30 species per cell). S is spatially correlated (Fig 2b): we found positive spatial autocorrelation at distances of 0–40 km and negative spatial autocorrelation starting at large distances (90 km).

The four environmental descriptors are also spatially structured. However, whereas mean annual temperature and annual precipitation vary at a relatively broad spatial scale, creating large gradients with positive autocorrelation at distances up to 60–70 km (S1a and S1b Fig and S3a and S3b Fig), both human population density and the percentage of natural and semi-natural areas vary at a relatively fine spatial scale with positive autocorrelation only at distances up to 20–30 km (S1c and S1d Fig and S3c and S3d Fig).

### Patterns of plant invasion explained by climate and human pressures

**Fig 3:**
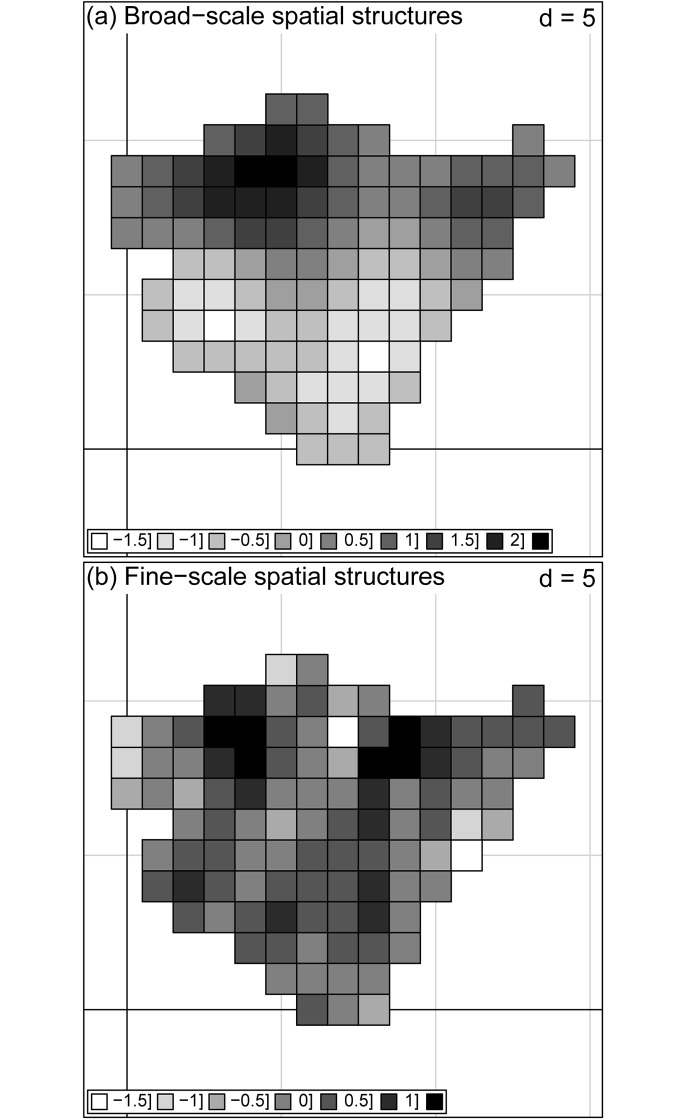
Patterns of invasive alien plant species per cell (S) in the Basque Country at broad (a) and fine (b) spatial scales. The maps represent fitted scores (*n* = 104) of RDA canonical axes modeling broad-scale (adjusted-*R*^2^ = 50.8% of the total variance in S) and fine-scale spatial structures (adjusted-*R*^2^ = 20.1% of the total variance in S). Distance (d) is in units of 10 km. See the environmental analysis of these spatial patterns in Tables 2 and 3.

We dissected the pattern in S (Fig 2) into two spatial components. The broad-scale spatial component of S represented *R*^2^ = 50.8% of the total variance (*F* = 20.2; *p* < 0.01) and is mapped in Fig 3a. In a posterior linear regression, mean annual temperature and annual precipitation explained 65.7% of the spatially structured variation in this RDA canonical axis (Table 2). Hence spatially structured climate descriptors explained *R*^2^ = (0.508 x 0.657) x 100 = 33.4% of the total variance in the response S. The fine-scale spatial component of the invasive plant richness represented *R*^2^ = 20.1% of the total variance in S (*F* = 6.2; *p* < 0.01) and is mapped in Fig 3b. In a posterior linear regression, spatially structured natural log-transformed human population density and percentage of natural and semi-natural areas explained 21.9% of the variation in this RDA canonical axis (Table 3). Consequently, spatially structured human pressure descriptors explained *R*^2^ = (0.201 x 0.219) x 100 = 4.4% of the total variance in S.

**Table 2:** RDA canonical axis modeling broad-scale spatial structures.

**Source**	**d.f.**	**SS**	**MS**	***F***	***p*-value**
ANOVA table:					
Mean annual temperature	1	48.90	48.90	158.5	0.000
Annual precipitation	1	8.60	8.60	27.9	0.000
Annual precipitation^2^	1	4.73	4.73	15.3	0.000
Residuals	101	31.15	0.31		
*S* = 0.56 (on 101 d.f.); adjusted-*R*^2^ = 65.7					
**Estimates of parameters:**					
**Term**	**Coefficient**	**SE Coef.**	***t***	***p*-value**	
Mean annual temperature	5.64	0.62	9.1	0.000	
Annual precipitation	3.23	0.62	5.2	0.000	
Annual precipitation^2^	-2.18	0.56	-3.9	0.000	

Regression analysis results of broad-scale spatial variation in the number of invasive alien plant species in the Basque Country (S) on climate descriptors. Note that the response is an RDA canonical axis modeling broad-scale spatial structure that explained 50.8% of the total variance in S (see a map for this axis in Fig 3a). In this model, mean annual temperature and annual precipitation (in the form of first- and second-order polynomial terms) significantly explained 65.7% of variation in the RDA canonical axis. Grain surface was not significant; the habitat use descriptors percentage of natural and semi-natural areas and human population density were not significant.

**Table 3:** RDA canonical axis modeling fine-scale spatial structures.

**Source**	**d.f.**	**SS**	**MS**	***F***	***p*-value**
ANOVA table:					
Population density	1	4.85	4.85	17.5	0.000
Population density^2^	1	1.74	1.74	6.3	0.014
Natural and semi-natural areas	1	2.32	2.32	8.4	0.005
Residuals	101	28.01	0.28		
*S* = 0.53 (on 101 d.f.); adjusted-*R*^2^ = 21.9					
**Estimates of parameters:**					
**Term**	**Coefficient**	**SE Coef.**	***t***	***p*-value**	
Population density	1.66	0.56	3.0	0.004	
Population density^2^	1.60	0.54	3.0	0.004	
Natural and semi-natural areas	-1.64	0.57	-2.9	0.005	

Regression analysis results of fine-scale spatial variation in the number of invasive alien plant species in the Basque Country (S) on human pressure descriptors. Note that the response is an RDA canonical axis modeling fine-scale spatial structure that explained 20.1% of the total variance in S (see a map for this axis in Fig 3b). In this model, population density (in the form of first- and second-order polynomial terms) and percentage of natural and semi-natural areas significantly explained 21.9% of variation in the RDA canonical axis. Grain surface was not significant. The climatic descriptors mean annual temperature and annual rainfall were not significant.

In summary, the fraction of S (Fig 2) that is spatially structured (as described by the PCNMs spatial variables) represented *R*^2^ = 50.8 (broad scale) + 20.1 (fine scale) = 70.9% (Fig 3). The fraction of S that was explained by spatially structured environmental variables added up to *R*^2^ = 33.4 (broad scale) + 4.4 (fine scale) = 37.8% (Tables 2 and 3), which indicates the presence of a marked environmentally induced spatial component in S. Mean annual temperature at a broad scale, and similar contributions of human population density and percentage of natural and semi-natural areas at a fine scale, were the most important environmental factors, which explains the location of the within-region S hot spots (Fig 3) in the warmest and highly populated northern areas of the Basque Country.

### Species-specific responses to climate and human pressures

**Fig 4:**
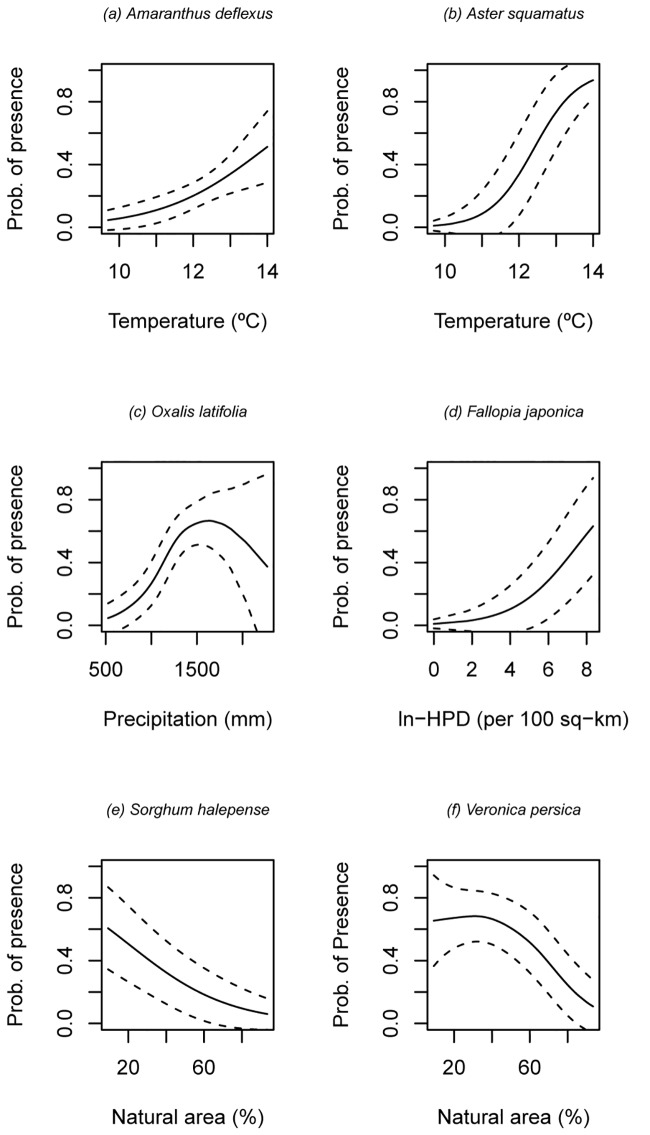
Fitted Generalized Additive Mixed Models for six example species, with 95% confidence bands. The smoothers show the probability of occurrence with increasing mean annual temperature (Temperature, a–b), annual precipitation (Precipitation, c), natural log-transformed human population density (ln-HPD, d) and percentage of natural and semi-natural areas (Natural area) in a given UTM cell (e–f). See Table 4 for statistical details.

Significant GAMM models were fitted for 36 out of 37 tested species (Table 4; Fig 4), with average adj.-*R*^*2*^ = 35%. *Dittrichia viscosa* was the only tested species whose presence seems to be independent of the considered environmental descriptors. The relationship between species probability of presence and mean annual temperature was significant for 28 species (76% of the tested species) and was always monotonic and increasing (either curvilinear concave or sigmoid-shaped; see examples in Fig 4a and 4b). Annual precipitation was significant only for 13 species (35%), with most relationships between species probability of presence and precipitation being hump-shaped (Fig 4c) or nearly so. Natural log-transformed human population density was significant for five species (13%), with all relationships being always increasing and curvilinear concave (Fig 4d). Finally, the relationships between species probability of presence and percentage of natural and semi-natural areas were significant for 21 species (57%); these relationships were always monotonic and decreasing, and either curvilinear concave or curvilinear convex (see examples in Fig 4e and 4f). Overall, 31 out of 37 frequent invasive alien species seem to be sensitive to either temperature, precipitation or both. In other words, the null hypothesis of no relationship between species occurrence and climatic descriptors was rejected for all species in Table 4 except *Amarathus retroflexus*, *Dittrichia viscosa*, *Fallopia japonica*, *Medicago sativa*, *Sorghum halepense* and *Veronica persica* (16% of tested species). Similarly, the null hypothesis of no relationship between species occurrence and human pressure descriptors was rejected for all species in Table 4 except *Amaranthus deflexus*, *A*. *hybridus*, *Arundo donax*, *Aster squamatus*, *Chenopodium ambrosioides*, *Cortaderia selloana*, *Dittrichia viscosa*, *Echinochloa crus-galli*, *Crocosmia x crocosmiiflora*, *Oenothera rosea*, *Solanum chenopodioides*, *Lepidium virginicum* and *Lonicera japonica* (35% of tested species). On the whole, mean annual temperature was the most influential environmental descriptor.

**Table 4:** Generalized Additive Mixed Modeling results for the 37 most frequent invasive alien plant species in the Basque Country (northern Spain).

			Mean annual temperature			Annual precipitation			Natural and semi-natural areas			Human Population Density		
Species	Relative frequency	*R*^2^ (adj.)	*F* (edf)	*p*-value	SR	*F* (edf)	*p*-value	SR	*F* (edf)	*p*-value	SR	*F* (edf)	*p*-value	SR
*Amaranthus blitum blitum*	0.29	0.451	-	-	·	4.9 (2.5)	0.006	SS (+)	6.6 (1.6)	0.004	X (-)	6.1(1.0)	0.016	C(+)
*Amaranthus deflexus*	0.23	0.074	8.0 (1.0)	0.007	C (+)	-	-	·	-	-	·	-	-	·
*Amaranthus hybridus*	0.44	0.227	5.7 (2.1)	0.004	SS (+)	-	-	·	-	-	·	-	-	·
*Amaranthus retroflexus*	0.38	0.082	-	-	·	-	-	·	3.8 (2.3)	0.023	X (-)	-	-	·
*Artemisia verlotiorum*	0.23	0.238	5.9 (1.0)	0.017	C (+)	-	-	·	9.0 (1.0)	0.003	C (-)	-	-	·
*Arundo donax*	0.27	0.512	16.9 (1.0)	0.000	SS (+)	-	-	·	-	-	·	-	-	·
*Aster squamatus*	0.49	0.323	9.8 (1.0)	0.002	SS (+)	-	-	·	-	-	·	-	-	·
*Bidens aurea*	0.28	0.449	16.0 (1.0)	0.000	C (+)	3.8 (2.4)	0.020	H	4.8 (1.7)	0.015	X (-)	-	-	·
*Bromus catharticus*	0.39	0.454	12.0 (1.9)	0.000	SS (+)	-	-	·	7.0 (1.0)	0.010	C (-)	-	-	·
*Buddleja davidii*	0.36	0.527	6.3 (6.0)	0.001	H	-	-	·	-	-	·	15.4 (1.0)	0.000	C(+)
*Centranthus ruber*	0.41	0.417	6.2 (1.0)	0.015	C (+)	4.1 (2.4)	0.014	H	6.2 (1.0)	0.015	C (-)	-	-	·
*Chenopodium ambrosioides*	0.31	0.208	13.2 (1.0)	0.000	C (+)	-	-	·	-	-	·	-	-	·
*Conyza bilbaoana*	0.64	0.383	-	-	·	6.2 (3.0)	0.001	H	16.5 (1.8)	0.000	X (-)	-	-	·
*Conyza sumatrensis*	0.60	0.404	8.1 (1.0)	0.005	C (+)	-	-	·	4.5 (2.7)	0.007	X (-)	-	-	·
*Coronopus didymus*	0.40	0.216	5.5 (1.0)	0.021	C (+)	-	-	·	3.1 (2.2)	0.044	X (-)	-	-	·
*Cortaderia selloana*	0.53	0.437	11.1 (1.0)	0.001	SS (+)	-	-	·	-	-	·	-	-	·
*Crocosmia x crocosmiiflora*	0.25	0.437	13.4 (1.8)	0.000	SS (+)	7.1 (1.7)	0.003	H	-	-	·	-	-	·
*Cymbalaria muralis*	0.38	0.409	5.5 (1.0)	0.021	C (+)	3.5 (2.4)	0.025	H	-	-	·	8.8 (1.0)	0.004	C(+)
*Cyperus eragrostis*	0.47	0.670	6.8 (1.0)	0.010	C (+)	10.3 (2.2)	0.000	SS (+)	6.5 (2.5)	0.001	X (-)	-	-	·
*Datura stramonium*	0.38	0.193	4.8 (1.0)	0.003	C (+)	-	-	·	3.0 (2.2)	0.049	X (-)	-	-	·
*Dittrichia viscosa*	0.35	0.000	-	-	·	-	-	·	-	-	·	-	-	·
*Echinochloa crus-galli*	0.46	0.314	8.7 (1.7)	0.001	SS (+)	-	-	·	-	-	·	-	-	·
*Erigeron karvinskianus*	0.38	0.596	13.9 (1.0)	0.000	C (+)	3.8 (3.5)	0.010	H	9.5 (1.0)	0.003	C (-)	-	-	·
*Fallopia japonica*	0.23	0.247	-	-	·	-	-	·	-	-	·	8.9 (1.0)	0.003	C(+)
*Lepidium virginicum*	0.34	0.313	6.6 (2.1)	0.002	SS (+)	-	-	·	-	-	·	-	-	·
*Lonicera japonica*	0.28	0.328	13.9 (1.0)	0.000	C (+)	-	-	·	-	-	·	-	-	·
*Medicago sativa*	0.45	0.089	-	-	·	-	-	·	3.9 (1.9)	0.025	X (-)	-	-	·
*Oenothera rosea*	0.25	0.211	3.3 (2.2)	0.038	SS (+)	-	-	·	-	-	·	-	-	·
*Oxalis latifolia*	0.43	0.506	11.1 (1.0)	0.001	C (+)	3.1 (2.7)	0.035	H	4.7 (3.6)	0.002	X (-)	-	-	·
*Paspalum dilatatum*	0.53	0.662	5.3 (2.1)	0.006	SS (+)	3.6 (2.5)	0.024	H	3.6 (2.5)	0.024	X (-)	-	-	·
*Paspalum distichum*	0.41	0.676	21.3 (1.0)	0.000	C (+)	-	-	·	3.9 (3.0)	0.011	X (-)	12.3 (1.0)	0.001	C(+)
*Platanus hispanica*	0.34	0.490	7.4 (1.0)	0.008	C (+)	3.5 (2.0)	0.036	H	8.4 (1.0)	0.005	C (-)	-	-	·
*Robinia pseudoacacia*	0.56	0.458	-	-	·	5.2 (3.4)	0.002	H	6.5 (2.6)	0.001	X (-)	-	-	·
*Solanum chenopodioides*	0.21	0.230	10.8 (1.0)	0.001	C (+)	-	-	·	-	-	·	-	-	·
*Sorghum halepense*	0.37	0.144	-	-	·	-	-	·	8.6 (1.0)	0.004	C (-)	-	-	·
*Sporobolus indicus*	0.50	0.597	4.2 (1.7)	0.026	SS (+)	4.9 (3.0)	0.003	H	9.0 (1.0)	0.003	C (-)	-	-	·
*Veronica persica*	0.60	0.139	-	-	·	-	-	·	5.1 (2.1)	0.007	X (-)	-	-	·

The fixed parts of six example species models are shown in Fig 4. *edf* = estimated degrees of freedom; SR = shape of relationship; X = curvilinear convex; C = curvilinear concave; SS = sigmoid-shaped; H = hump-shaped; (+) = increasing; (-) = decreasing.

On average, annual species (relative frequency = 0.42) are slightly more frequent than perennial species (relative frequency = 0.37). However, the climate and human pressure descriptors (mean annual temperature, annual precipitation, percentage of natural and semi-natural areas, and human population density) explain higher proportion of variance for perennials (average *R*^2^ across species = 0.40) than for annuals (average *R*^2^ species = 0.26). In general, the presence-absence of both annuals and perennials tend to display sigmoid-shaped (positive) or curvilinear concave (also positive) relationships with mean annual temperature. By contrast, whereas the presence-absence of annuals tends to be little affected by annual precipitation, the presence-absence of perennials tends to display hump-shaped relationships with annual precipitation, with optima located at intermediate values of this environmental descriptor. Similarly, the presence-absence of both annual and perennial species tends to display curvilinear (-) relationships with the percentage of natural and semi-natural areas. By contrast, whereas the presence-absence of annuals tends to be little affected by human population density, the presence-absence of perennials tends to display curvilinear concave (+) relationships with this variable.

Regarding the broadest phylogenetic groups (monocots vs. eudicots), the climate and human pressure descriptors explain more variance for monocots (average *R*^2^ across species = 0.49) than for eudicots (average *R*^2^ across species = 0.30); this can be explained because most monocots are perennials (90%) while only 60% of eudicots are perennials. However, the form and frequency of the relationships between the presence-absence of both monocots and eudicots and the climate and habitat use descriptors are similar and no particular pattern can be discerned.

## Discussion

Modeling the spatial distribution of the number (per UTM cell) of invasive alien plant species (invasive plant richness S) at two spatial scales allowed us to uncover the pattern of alien plant invasion and thereby to identify invasion hot spots in the warmest and highly populated northern areas of the Basque Country. We also disentangled the effect of climate and human pressure descriptors on S at broad and fine scales. Species-specific GAMMs showed that most tested species are sensitive to climate descriptors (mean annual temperature, annual precipitation or both) and human pressure descriptors (either percentage of natural and semi-natural areas or human population density), with similar and consistent responses that, overall, seem to shape S as an integrated response.

### The spatial structure of the invasive plant richness is strongly environmentally induced

The broad-scale spatial component of S, which is the most important spatial component, was, to a large extent (65.7%), explained by mean annual temperature and annual precipitation. The fine-scale spatial component of S was partially explained (21.9%) by human population density and percentage of natural and semi-natural cover. Human population density and human settlements are factors positively correlated with alien plant abundance and propagule pressure [10, 40]. Moreover, population density was correlated in our study with land use descriptors such as the percentage of cultivated areas and of infrastructural areas and, therefore, it can be considered as a good proxy of disturbance. It is well known that human mediated disturbances provide opportunities for invaders to spread [41], form “corridor pathways” [42, 43], and can increase above- and below-ground resource availability [44]. Although a number of studies have also reported a positive correlation between alien plant species richness and temperature, precipitation and/or human activity (e.g., [45–48]), they do not specifically address the spatial scales at which these spatially structured variables operate. However, in our study, by performing PCNM and variation partitioning analyses [27, 28] we were able to capture environment-induced spatial structures at different scales [49]. Because the organisms are constrained by their physiological tolerances to climatic factors, it is widely accepted that climate governs species distributions at broad biogeographical scales [16, 17, 50, 51]. Our results support the idea that the species distributions are hierarchically structured and that climatic variables are large-scale determinants, followed by land cover or disturbance predictors at smaller resolutions [16, 17]. Modeling the corresponding spatial structures separately at different scales is of valuable use for ecologists, though not largely used (but see [49, 52–55]). To our knowledge, this is the first study that specifically addresses this issue in alien plant invasion spatial patterns.

In our study the unexplained variance in the spatially structured invasive species richness amounted to 34.3% at a broad scale and 78.1% at a fine scale. This fact, in part, might be the outcome of the sampling bias that is likely to affect the data collection compiled in databases, due to variable sampling effort among different areas. Moreover, other factors that have not been taken into account in this study could explain part of the unexplained variance. For instance, at a broad scale, the suitability of our model might be reduced by the fact that many invasive species have not achieved their potential distribution range yet [56, 57] or that they differ in the residence time [58]. In addition, the response of some species that preferably inhabit azonal aquatic habitats such as riverbeds or estuaries (e.g. *Baccharis halimifolia*) or other saline habitats, such as dunes and cliffs, might be difficult to model at this scale. In fact, these coastal habitats have been identified as very prone to invasion [59, 60], showing a high proportion of the invasive flora of the territory.

At a fine spatial scale, the spatial structure we found might indirectly reflect dispersal issues, on top of spatially structured environmental factors. Propagule pressure [61] or spatial structure induced by species dispersal kernels could explain part of the unexplained variance. The integration of other factors such as biotic interactions and dispersal mechanisms into bioclimatic models might contribute to improving the accuracy of models [14, 62].

### Species-specific responses to climate and human pressure descriptors

Most tested species responded to climate predictors (84%), particularly to temperature (76%), and to human pressure descriptors (65%). Importantly, all monotonic species responses had the same sign for each predictor, i.e. the probability of presence of invasive plants always increased as mean temperature and human population density increased, and as the percentage of natural and semi-natural areas decreased. Moreover, the relationship between species probability of presence and rainfall, when significant, was hump-shaped in most cases and the optimum in these relationships was nearly always found to be around 1500 mm per year. On the other hand, the average adj.-*R*^*2*^ = 35% found for these fitted GAMMs (Table 4; Fig 4) is clearly comparable with the adj.-*R*^*2*^ = 34 + 4 = 38% that was found in the PCNM analysis.

Some species such as *Cortaderia selloana*, *Arundo donax* and *Lonicera japonica* responded uniquely to climate descriptors, mainly to mean annual temperature, and the distribution of these species is typically restricted to areas located at low altitudes that lack winter frost in the Basque Country. However, the distribution of 51% of the tested species was better explained by a combination of climate and human pressure descriptors. Most successful invaders in the Basque Country have actually been introduced from warm and subtropical bioclimates and are frequently associated with human-induced disturbances [63]. For instance, common species like *Centranthus ruber* and *Erigeron karvinskianus*, though highly associated with warm climates [64], also typically inhabit urban walls and rocky roadsides. Several ruderal speciessuch as *Bidens aurea*, *Bromus catharticus*, *Conyza sumatrensis* and *Oxalis latifolia* are at the same time thermophilous species distributed at low latitudes [64, 65]. Likewise, alien grasses that are very abundant in the region, such as *Paspalum dilatatum* and *Sporobolus indicus*, have a neotropical origin [66, 67] and invade mainly disturbed trampled grasslands in the Basque Country [20].

Despite the above general pattern, five species proved to be sensitive only to human pressure constraints: *Sorghum halepense*, *Veronica persica*, *Medicago sativa*, *Amaranthus retroflexus* and *Fallopia japonica*. Most of them are segetal or ruderal species that are known to be widespread throughout differing bioclimatic regions with dissimilar temperature and precipitation regimes [65, 67]. The fitted models for these species had a rather low goodness of fit (*R*^2^ ≤ 0.1). One single species, *Dittrichia viscosa*, did not respond to any of the considered descriptors, possibly because in the Basque Country it has a typical row-shaped distribution along some of the main highways. This last result indicates that plant invasions may be driven by species-specific ecological requirements, which might also be taken into account when modeling invasive plant species distribution. Although invasion hot spots are major targets for managers, particular individual species might represent significant threats to native ecosystems as well. Therefore, in order to identify areas for prevention and control of particularly aggressive species, it might be necessary to consider additional alternative predictors, such as presence of certain types of habitats [60], at least if more conservative attempts fail.

## Conclusion and Management Implications

Consistent results from two complementary statistical approaches (PCNM method and GAMM) allowed us to show that the spatial structure of alien plant invasion in the Basque Country is strongly environmentally induced, and to disentangle the scale-specific importance of corresponding environmental factors. These findings will be useful for performing simulations of plant invasions but also for highlighting the importance of undertaking multi-scale approaches in such simulations to better understand environmental limitations to the spreading of invasive species. In the Basque Country, regional-scale predictions on hot spots or on individual species could be made on a climatic basis using data obtained at broad scales. In contrast, local conservation policy planning might focus on local-scale predictions based on human pressure factors analyzed at a finer scale resolution. Likewise, for particularly aggressive species control and prevention, individual species models may help in taking decisions on the necessary scale to be considered according to the most influential environmental factor for that particular species. This study thus may help optimize conservation efforts across administrations by providing information on the appropriate scale for data resolution (grain) and for prediction parameterization.

## Supporting Information

S1 Fig Environmental heterogeneity in the Basque Country, northern Spain. Maps of (a) mean annual temperature, (b) annual precipitation, (c) log-transformed human population density and (d) percentage of natural and semi-natural areas. Lower bounds must be interpreted as less or equal than printed value: for example, the white quadrats in the map for annual precipitation (b) indicate that annual rainfall is less or equal than 1000 mm. Distance (d = 5) is in units of 10 km.
(PDF)

S2 Fig Bivariate plots with smooth curves, histograms and Kendall correlations. Bivariate plots, histograms and Kendall correlations for the climate and human pressure constraints used as explanatory variables for the number of invasive alien plant species and species’ individual responses. Human population density was log-transformed to achieve symmetry and hence make it more amenable to linear modeling. NaturPerc = percentage of natural and semi-natural areas; T = mean annual temperature (°C); lnPopDen = natural log-transformed human population density per 100 km^2^; P = annual precipitation (mm); Grain surface in km^2^.
(PDF)

S3 Fig Spatial correlograms. Spatial correlograms for mean annual temperature, annual precipitation, human population density and percentage of natural and semi-natural areas. These correlograms can be compared with the maps in S2 Fig. Mean annual temperature and annual precipitation vary at (relatively) broad spatial scales, whereas human population density and the percentage of natural and semi-natural areas vary at (relatively) fine spatial scales. Distance is in units of 10 km.
(PDF)

S4 Fig Principal Coordinates of Neighbor Matrices. Sixteen PCNM spatial variables with positive spatial correlation that significantly explained spatial structures in the species number of invasive alien plants in the Basque Country. According to the size of the patterns, the first five PCNMs (1, 2, 4, 6, and 8) were selected to model spatial variation at a broad scale; the other PCNMs were used to model spatial variation at a fine scale. In order to learn more on the use of these spatial templates, see S2 File in the Supporting Information (R code).
(PDF)

S1 File Dataset used in this study. The full data of species occurrences and descriptors in UTM cells.
(TXT)

S2 File R code. Together with S1 File, this R code allows the full replication of our statistical analysis.
(R) Click here for additional data file.

S1 Table Frequencies of studied alien species. List of invasive alien plant species with their relative frequencies in the Basque Country region, northern Spain. Species with relative frequency greater than 20% (grey) were tested by means of generalized additive mixed modeling (GAMM).
(PDF)
