# Supplementary material for: Climate and Human Pressure Constraints Co-Explain Regional Plant Invasion at Different Spatial Scales
Source: PLoS One. 2016 Oct 14;11(10):e0164629. doi: 10.1371/journal.pone.0164629 (PMC5065173; doi:10.1371/journal.pone.0164629)
Supplement: S1 Fig — Maps of (a) mean annual temperature, (b) annual precipitation, (c) log-transformed human population density and (d) percentage of natural and semi-natural areas. Lower bounds must be interpreted as less or equal than printed value: for example, the white quadrats in the map for annual precipitation (b) indicate that annual rainfall is less or equal than 1000 mm. Distance (d = 5) is in units of 10 km. (PDF) [file pone.0164629.s001.pdf]

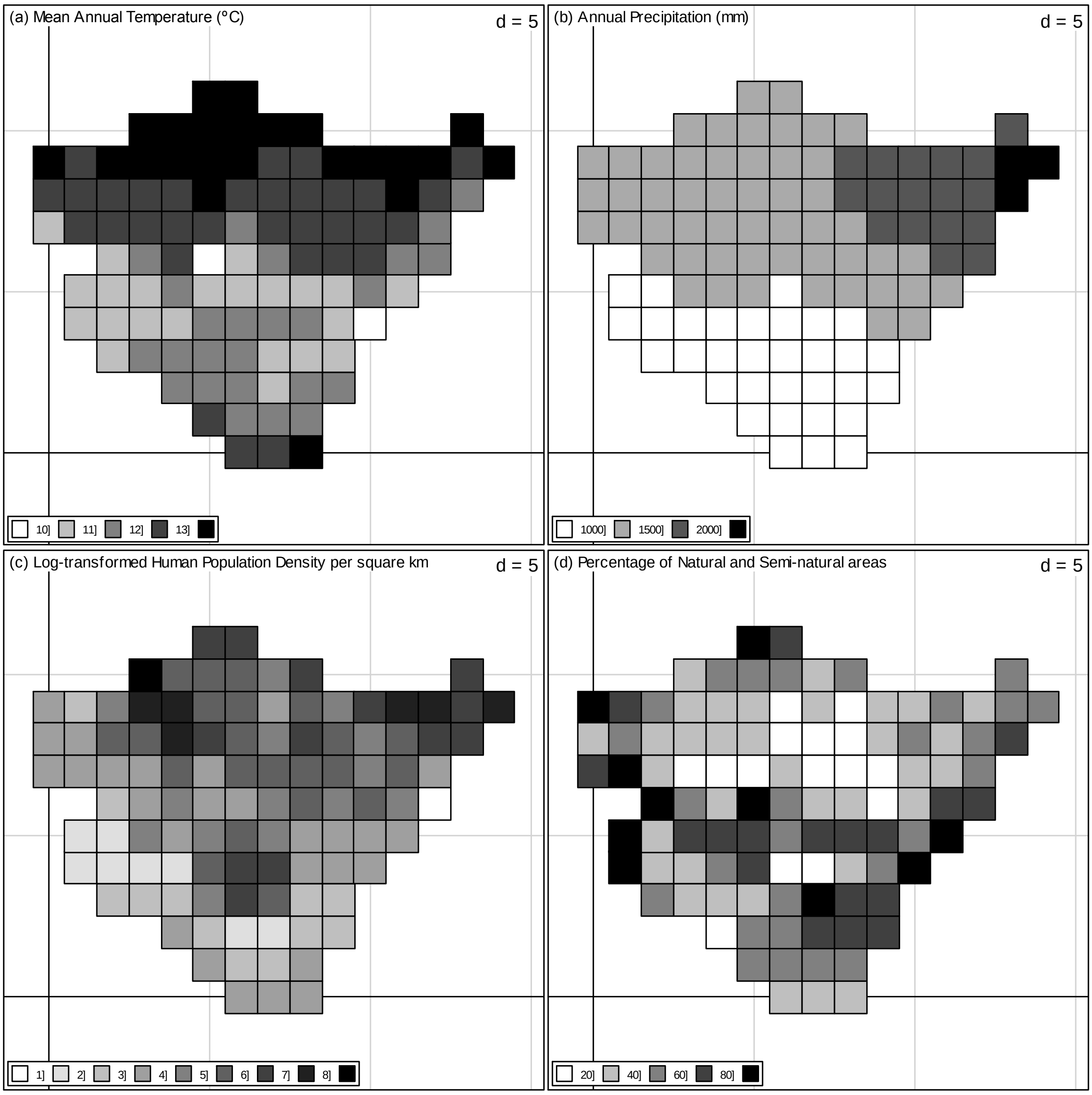

S1 Fig. Environmental heterogeneity in the Basque Country, northern Spain. Maps of (a) mean annual temperature, (b) annual precipitation, (c) log-transformed human population density and (d) percentage of natural and semi-natural areas. Lower bounds must be interpreted as less or equal than printed value: for example, the white quadrats in the map for annual precipitation (b) indicate that annual rainfall is less or equal than 1000 mm. Distance (d = 5) is in units of 10 km.
