# Supplementary material for: Climate and Human Pressure Constraints Co-Explain Regional Plant Invasion at Different Spatial Scales
Source: PLoS One. 2016 Oct 14;11(10):e0164629. doi: 10.1371/journal.pone.0164629 (PMC5065173; doi:10.1371/journal.pone.0164629)
Supplement: S2 Fig — Bivariate plots, histograms and Kendall correlations for the climate and human pressure constraints used as explanatory variables for the number of invasive alien plant species and species’ individual responses. Human population density was log-transformed to achieve symmetry and hence make it more amenable to linear modeling. NaturPerc = percentage of natural and semi-natural areas; T = mean annual temperature (°C); lnPopDen = natural log-transformed human population density per 100 km2; P = annual precipitation (mm); Grain surface in km2. (PDF) [file pone.0164629.s002.pdf]

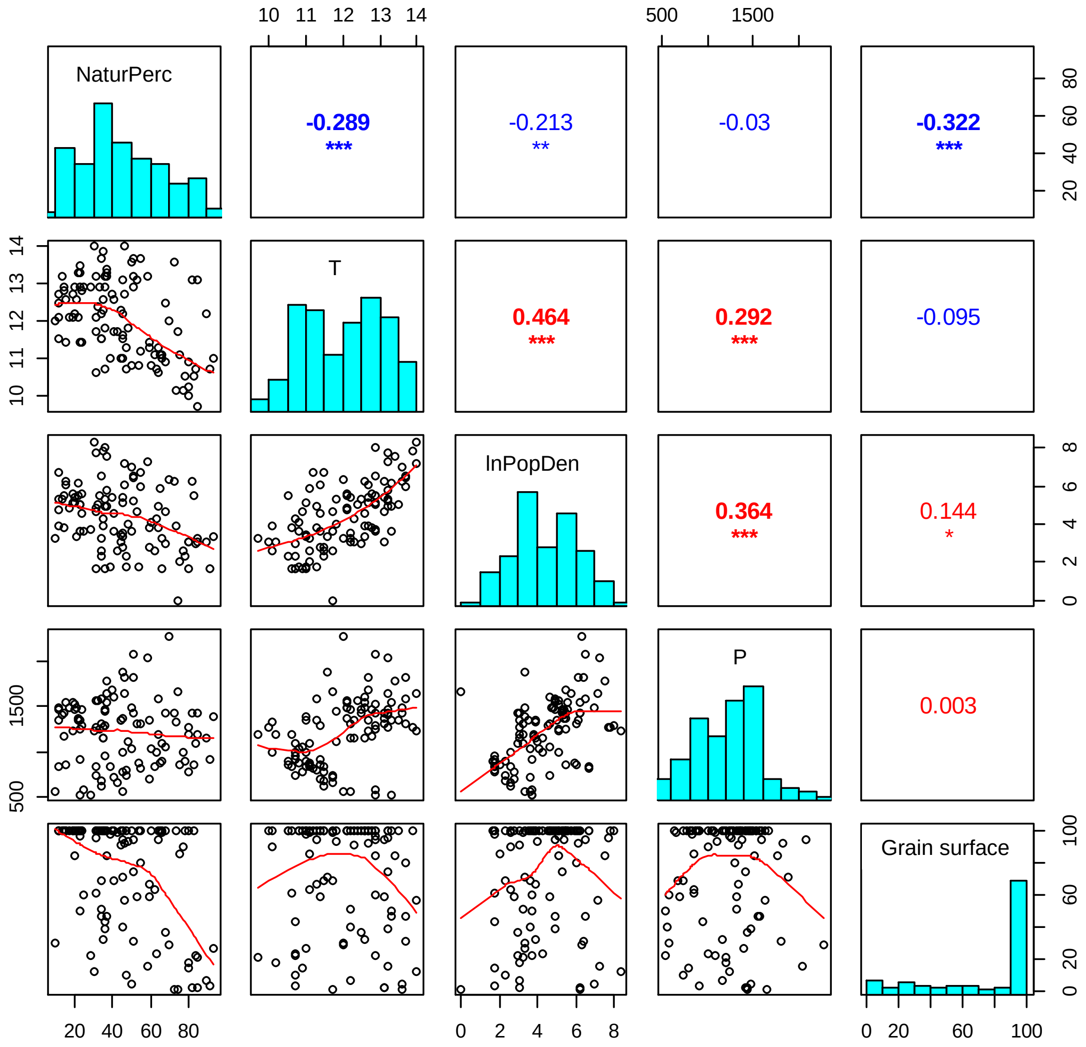

S2 Fig. Bivariate plots, histograms and Kendall correlations for the climate and human pressure constraints used as explanatory variables for the number of invasive alien plant species and species' individual responses. Human population density was log-transformed to achieve symmetry and hence make it more amenable to linear modelling. NaturPerc = percentage of natural and semi-natural areas; T = mean annual temperature (ºC); lnPopDen = natural log-transformed human population density per 100 km^2; P = annual precipitation (mm); Grain surface in km^2.
