# Supplementary material for: Climate and Human Pressure Constraints Co-Explain Regional Plant Invasion at Different Spatial Scales
Source: PLoS One. 2016 Oct 14;11(10):e0164629. doi: 10.1371/journal.pone.0164629 (PMC5065173; doi:10.1371/journal.pone.0164629)
Supplement: S3 Fig — Spatial correlograms for mean annual temperature, annual precipitation, human population density and percentage of natural and semi-natural areas. These correlograms can be compared with the maps in S2 Fig. Mean annual temperature and annual precipitation vary at (relatively) broad spatial scales, whereas human population density and the percentage of natural and semi-natural areas vary at (relatively) fine spatial scales. Distance is in units of 10 km. (PDF) [file pone.0164629.s003.pdf]

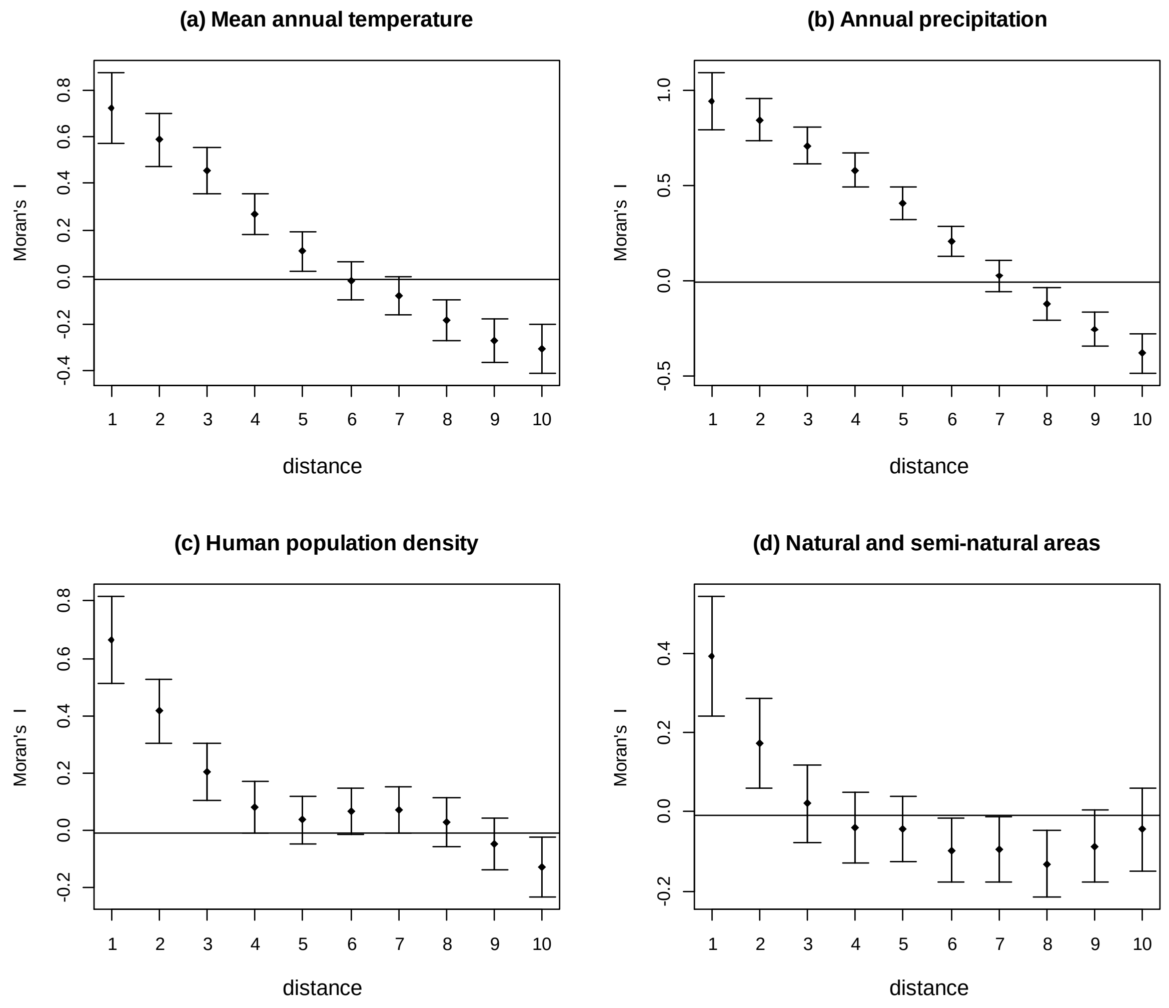

S3 Fig. Spatial correlograms for mean annual temperature, annual precipitation, human population density and percentage of natural and semi-natural areas. These correlograms can be compared with the maps in S2 Fig. Mean annual temperature and annual precipitation vary at (relatively) broad spatial scales, whereas human population density and the percentage of natural and semi-natural areas vary at (relatively) fine spatial scales. Distance is in units of 10 km.
