# Supplementary material for: Climate and Human Pressure Constraints Co-Explain Regional Plant Invasion at Different Spatial Scales
Source: PLoS One. 2016 Oct 14;11(10):e0164629. doi: 10.1371/journal.pone.0164629 (PMC5065173; doi:10.1371/journal.pone.0164629)
Supplement: S4 Fig — Sixteen PCNM spatial variables with positive spatial correlation that significantly explained spatial structures in the species number of invasive alien plants in the Basque Country. According to the size of the patterns, the first five PCNMs (1, 2, 4, 6, and 8) were selected to model spatial variation at a broad scale; the other PCNMs were used to model spatial variation at a fine scale. In order to learn more on the use of these spatial templates, see S2 File in the Supporting Information (R code). (PDF) [file pone.0164629.s004.pdf]

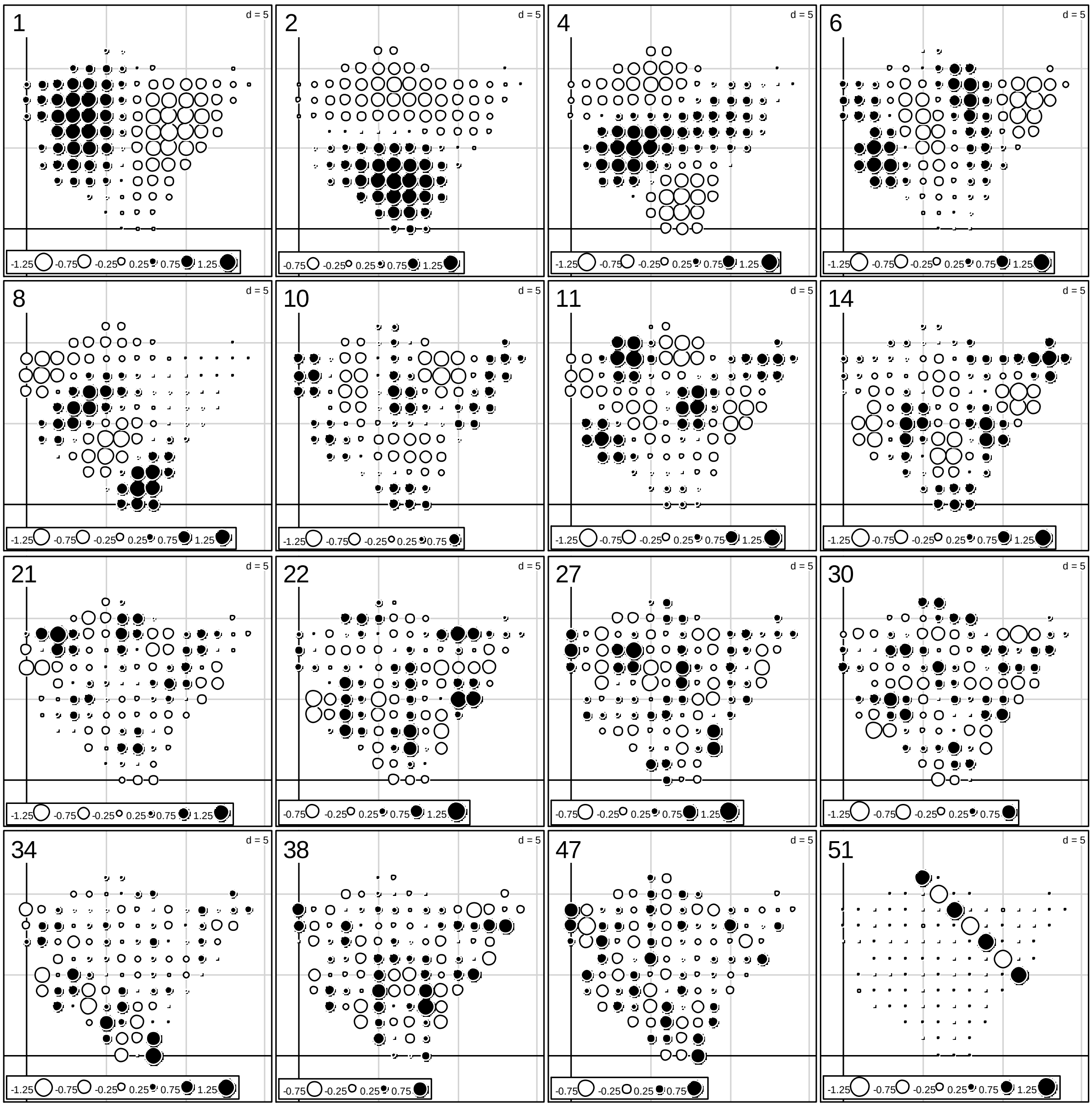

S4 Fig. Sixteen PCNM spatial variables with positive spatial correlation that significantly explained spatial structures in the species number of invasive alien plants in the Basque Country. According to the size of the patterns, the first five PCNMs (1, 2, 4, 6, and 8) were selected to model spatial variation at a broad scale; the other PCNMs were used to model spatial variation at a fine scale. In order to learn more on the use of these spatial templates, see S2 File in the Supporting Information (R code).
