# Supplementary material for: Climate and Human Pressure Constraints Co-Explain Regional Plant Invasion at Different Spatial Scales
Source: PLoS One. 2016 Oct 14;11(10):e0164629. doi: 10.1371/journal.pone.0164629 (PMC5065173; doi:10.1371/journal.pone.0164629)
Supplement: S1 Table — List of invasive alien plant species with their relative frequencies in the Basque Country region, northern Spain. Species with relative frequency greater than 20% (grey) were tested by means of generalized additive mixed modeling (GAMM). (PDF) [file pone.0164629.s007.pdf]

S1 Table. List of invasive alien plant species with their relative frequencies in the Basque Country region, northern Spain. Species with relative frequency greater than 20% (grey) were tested by means of generalized additive mixed modeling (GAMM).

Species	Relative Frequency (%)
Abutilon theophrasti Medicus	6.7
Acacia dealbata Link	18.3
Acacia melanoxylon R. Br.	6.7
Ailanthus altissima (Miller) Swingle	12.5
Amaranthus albus L.	18.3
Amaranthus blitum L. subsp. *blitum*	28.8
Amaranthus bouchonii Thell.	16.3
Amaranthus cruentus L.	14.4
Amaranthus deflexus L.	23.1
Amaranthus graecizans L. *silvestris* (Vill.) Brenan	14.4
Amaranthus hybridus L.	44.2
Amaranthus hypochondriacus L.	12.5
Amaranthus powellii S. Watson	12.5
Amaranthus retroflexus L.	38.5
Arctotheca calendula L. Levyns	6.7
Artemisia verlotiorum Lamotte	23.1
Arundo donax L.	26.9
Aster squamatus (Sprengel) Hieron.	49
Baccharis halimifolia L.	14.4

Bidens aurea (Aiton) Sherff	27.9
Bidens frondosa L.	16.3
Bromus catharticus Vahl	39.4
Buddleja davidii Franchet	35.6
Carpobrotus edulis (L.) N.E. Br.	5.8
Centranthus ruber (L.) DC. subsp. *ruber*	41.3
Chenopodium ambrosioides L.	30.8
Coleostephus myconis (L.) Reichenb. fil.	9.6
Conyza bonariensis (L.) Cronq.	17.3
Conyza canadensis (L.) Cronq.	64.4
Conyza sumatrensis (Retz.) E. Walker	59.6
Coronopus didymus (L.) Sm.	40.4
Cortaderia selloana (Schult. & Schult. f.) Asch. & Graebn.	52.9
Crocosmia x *crocosmiiflora* (Lemoine) N.E. Br.	25
Cymbalaria muralis P. Gaertner, B. Meyer & Scherb. subsp. *muralis*	38.5
Cyperus eragrostis Lam.	47.1
Datura stramonium L.	38.5
Dichondra micrantha Urb.	3.8
Dittrichia viscosa (L.) W. Greuter subsp. *viscosa*	34.6
Duchesnea indica (Andrews) Focke	8.7
Echinochloa crus-galli (L.) Beauv.	46.2
Eragrostis virescens C. Presl	15.4
Erigeron karvinskianus DC.	38.5
Fallopia japonica (Houtt.) Ronse Decr.	23.1
Galinsoga quadriradiata Ruiz & Pavon	13.5

Gamochaeta coarctata (Wild.) Kerguélen	10.6
Helianthus tuberosus L.	8.7
Helianthus x laetiflorus Pers.	9.6
Impatiens balfourii Hooker fil.	10.6
Ipomoea indica (Burm.) Merr.	19.2
Juncus tenuis Willd.	7.7
Lepidium virginicum L.	33.7
Lonicera japonica Thunb.	27.9
Matricaria discoidea DC.	16.3
Matthiola incana (L.) R. Br. subsp. *incana*	14.4
Medicago sativa L. subsp. *sativa*	45.2
Oenothera drummondii Hooker subsp. *drummondii*	1.9
Oenothera glazioviana Micheli	17.3
Oenothera rosea L'Hér. ex Aiton	25
Oenothera x *fallax* Renner	3.8
Oxalis latifolia Kunth	43.3
Paspalum dilatatum Poiret	52.9
Paspalum distichum L.	41.3
Paspalum vaginatum Swartz	15.4
Phytolacca americana L.	11.5
Pinus pinaster Aiton	9.6
Platanus hispanica Mill. ex Münchh	33.7
Pterocarya stenoptera C. DC.	2.9
Robinia pseudoacacia L.	55.8
Senecio cineraria DC.	5.8

Senecio inaequidens DC.	8.7
Senecio mikanioides Otto ex Walpers	15.4
Setaria parviflora (Poir.) Kerguélen	11.5
Sisyrinchium angustifolium Mill.	5.8
Solanum chenopodioides Lam.	21.2
Soliva pterosperma (Juss.) Less.	3.8
Sonchus tenerrimus L.	9.6
Sorghum halepense (L.) Pers.	36.5
Spartina alterniflora Loisel.	6.7
Spartina patens (Ait.) Muhl.	6.7
Spartina x *townsendii* H. Groves & J. Groves	1.9
Sporobolus indicus (L.) R. Br.	50
Stenotaphrum secundatum (Walter) O. Kuntze	12.5
Tradescantia fluminensis Velloso	17.3
Tropaeolum majus L.	9.6
Verbena bonariensis L.	6.7
Veronica persica Poir.	59.6
Vinca difformis Pourret subsp. *difformis*	15.4
Xanthium spinosum L.	14.4
Xanthium strumarium L. subsp. *italicum* (Moretti) D. Löve	17.3
